# Nutritional performance of the tomato fruit borer, *Helicoverpa armigera* , on different tomato cultivars

**DOI:** 10.1093/jis/14.1.102

**Published:** 2014-08-01

**Authors:** Davoud Kouhi, Bahram Naseri, Ali Golizadeh

**Affiliations:** Department of Plant Protection, Faculty of Agriculture, University of Mohaghegh Ardabili, Ardabil, Iran

**Keywords:** nutritional indices, Noctuidae, Noctuidae, Noctuidae

## Abstract

The development and cultivation of tomato cultivars that are resistant to the tomato fruit borer, *Helicoverpa armigera* (Hübner) (Lepidoptera: Noctuidae), are very limited in Iran and other parts of the world because of the lack of information about resistant tomato cultivars to minimize the use of insecticides. Therefore, the present study was carried out to identify alternative methods to chemical control. Nutritional performance of the larval stages (fourth, fifth, and sixth instars) of *H. armigera* on fruit of eight tomato cultivars, including SUN 6108 f1, Rio grande UG, Korral, Super strain B, CH falat, Hed rio grande, Cal.JN3, and Super crystal, was studied under laboratory conditions. Fourth instars reared on CH falat and SUN 6108 f1 respectively showed the highest and lowest values of approximate digestibility. The highest values of efficiency of conversion of ingested food and efficiency of conversion of digested food of fifth instars were on Super strain B. The relative consumption rate and relative growth rate values of the sixth instars were the highest on Korral. The highest and lowest values of consumption index of sixth instars were on Super strain B and Hed rio grande, respectively. The efficiency of conversion of ingested food and efficiency of conversion of digested food values of whole larval instars were the highest on Hed rio grande and lowest on Rio grande UG. The results of nutritional indices indicated that Rio grande UG is an unsuitable host for *H. armigera* .

## Introduction

Tomato *(Ly copersicon esculentum* Mill. (Solanales: Solanaceae)) is one of the most important vegetables grown in many parts of the world because it is a good source of vitamins. During 2010-2011, 163,000 ha were under cultivation in Iran, with a total production of 5,887 million tons of tomatoes, and a yield of 36,189 Kg/ha ( Iran Ministry of Agriculture 2011 ). A wide range of insects attack tomato and are a major limiting factor in its successful cultivation and yield ( Ashok Kumar et al. 2009 ). Tomato is more susceptible to the pests’ attack than other vegetable crops, mainly because of its tenderness and softness. It is devastated by an array of pests; however, the major damage is caused by the tomato fruit borer, *Helicoverpa armigera* (Hübner) (Lepidoptera: Noctuidae) ( Sajjad et al. 2011 ). This insect is a highly polyphagous and serious pest that infests more than 100 plant species, including vastly planted, economically important crops such as cotton, maize, tobacco, pigeonpea, and chickpea ( Talekar et al. 2006 ). It causes widespread economic damage to tomato farms in Asia ( Srinivasan 1959 ; Singh and Singh 1975 ; Vattanatungum and Ruchtapakornchai 1978 ; Talekar et al. 1984 ). Controlling the insect pests with insecticides causes serious side effects, including development of insecticide resistance in the insects, pest resurgence, environmental pollution, and health hazards. The development and cultivation of *H. armigera-* resistant tomato cultivars are very limited in Iran and the world because of the lack of information about resistant tomato cultivars. Therefore, the present study was carried out to identify alternative methods to chemical control.

Different nutritive values of host plants can influence the rate of development of *H. armigera* larvae and so affect the population dynamics of this pest ( Ruan and Wu 2001 ). In addition, food consumption and use link plants attributes with insect performance ( Slansky 1990 ). Host plant resistance is an important tool in integrated pest management (IPM) ( Sajjad et al. 2011 ).

To determine the potential of different tomato cultivars to manage *H. armigera* populations, data on the effects of various cultivars on nutritional indices of the pest are necessary. In spite of the economic importance of *H. armigera* in tomato farms, to our knowledge, no published information exists on the nutritional indices of this pest on different tomato cultivars. Some related studies, however, have been conducted on the effects of host plants, apart from tomato cultivars, on nutritional indices of *H. armigera* ( Ashfaq et al. 2003 , Naseri et al. 2010 , Arghand et al. 2011 , Hemati et al. 2012 ). The objective of this study was to compare nutritional indices of *H. armigera* larvae on tomato cultivars. The results of this research can be used in IPM programs of *H. armigera* on tomato.

## Materials and Methods

### Tomato sources

Seeds of eight tomato *(L. esculentum)* cultivars, SUN 6108 f1, Rio grande UG, Super crystal, CH falat, Super strain B, Hed rio grande, Korral, and Cal.JN3, were obtained from the Plant and Seed Modification Research Institute, Karaj, Iran. The selected tomato cultivars are the most economically important cultivars grown in Iran and many other parts of the world. They were sown in the research field of the University of Mohaghegh Ardabili, located in Ardabil, Iran, in May 2011. For this study, the leaves of different tomato cultivars were used for feeding first and second larval instars, and the fruits were used for feeding third to sixth larval instars.

### Laboratory colony

*Helicoverpa armigera* larvae were originally collected from tomato fields of the Moghan region in Northwest Iran. The stock culture was initiated on different tomato cultivars in a growth chamber at 25 ± 1°C, 65 ± 5% RH, with a 16:8 L:D photoperiod.

### Experiments

Neonate larvae were collected from the stock cultures and divided into five replicates (10 larvae in each) and transferred into plastic containers (18.5 cm diam × 7.5 cm deep) with a hole covered by a mesh net for aeration; the containers held the fresh leaves of each tomato cultivar. The petioles of detached leaves were covered with water-soaked cotton to maintain freshness. Nutritional indices were determined using fourth to sixth instars because they are more easily measured than the primary instars. The first, second, and third instars were reared in groups until they reached the fourth instar, after which they were separated into individual plastic plates (8 cm diam × 1 cm deep) to avoid cannibalistic behavior. Sixth instars were kept in plastic tubes (1.5 cm diamx 5 cm deep) for prepupa-tion and pupation.

The gravimetric method ( Waldbauer 1968 ) was used to determine weight gain, food utilization, and feces produced. Nutritional indices were calculated on the dry weight basis. After measuring the weight of the fourth instars, they were introduced on the fruits of different tomato cultivars; the weights of the larvae were recorded daily before and after feeding until they finished feeding and reached the prepupal stage. The initial fresh fruits and the fruits and feces remaining at the end of each experiment were weighed daily. The quantity of food ingested was determined by subtracting the diet remaining at the end of each experiment from the total weight of fresh diet supplied. The weight of feces produced by the larvae was recorded daily. To obtain the dry weights of the fruits, feces, and larvae, 20 specimens from each cultivar were weighed, oven-dried (48 hr at 60°C), and then reweighed.

The formulas described by Waldbauer (1968) were used to determine nutritional indices; CI (consumption index), AD (approximate digestibility), ECI (efficiency of conversion of ingested food), ECD (efficiency of conversion of digested food), RCR (relative consumption rate), and RGR (relative growth rate):

$CI = \frac{E}{A}$

$AD = \frac{E-F}{E}$

$ECI = \frac{P}{E}$

$ECD = \frac{P}{E-F}$

$RCR = \frac{E}{A*T}$

$RGR = \frac{P}{A*T}$

in which, A = mean dry weight of insect over unit time, E = dry weight of food consumed, F = dry weight of feces produced, P = dry weight gain of insect, and T = duration of feeding period.

### Data analysis

Nutritional indices of *H. armigera* reared on different tomato cultivars were analyzed with one-way ANOVA using the statistical software Minitab 16 (Minitab, www.minitab.com ) to determine the similarities or significant differences. Statistical differences among the means were evaluated using the LSD test at α = 0.05. Before analysis, data were tested for normality.

A dendrogram of different tomato cultivars based on nutritional indices of whole larval instars (fourth, fifth, and sixth instars) of *H. armigera* on different cultivars of tomato was constructed after cluster analysis by Ward’s method using SPSS 16.0 statistical software (IBM, www.ibm.com ).

## Results

The results of the nutritional indices of fourth, fifth, and sixth, and whole larval instars of *H. armigera* are provided in Tables 1 , 2 , 3 , and 4 . Nutritional indices of fourth instars of *H. armigera* , except RCR, were significantly dif-different on tomato cultivars ( *P* < 0.01). The larvae reared on cultivar Cal.JN3 showed the highest values of ECI ( *F* = 3.41; df = 125; *P* < 0.01) and ECD ( *F* = 3.50; df = 125; *P* < 0.01). The lowest values of ECI and ECD were on Super crystal. The highest values of AD ( *F* = 10.02; df = 125; *P* < 0.01) and CI ( *F* = 2.94; df = 125; *P* < 0.01) were observed on cultivar CH falat. However, the lowest value of AD was on SUN 6108 f1. The larvae that fed on Korral and Super crystal respectively showed the highest and lowest RGR values ( *F* = 3.73; df = 125; *P* < 0.01). However, the lowest value of CI was on cultivar Hed rio grande (Ta-( Table 1 ). The highest values of RCR (F = 2.70; df = 125; *P* < 0.05) and RGR (F = 4.64; df = 125; *P* < 0.01) of fifth instars were observed on Korral. The larvae that fed on cultivars Rio grande UG and Korral had respectively the highest and lowest values of AD ( *F* = 36.21; df = 125; *P* < 0.01) ( Table 2 ).

**Table 1.**
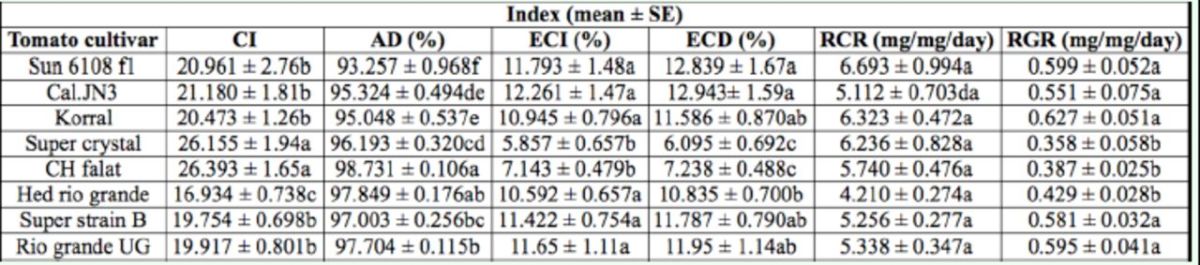
Nutritional indices of fourth instar larvae of *Helicoverpa armigera* on different tomato cultivars.

The means followed by different letters in the same columns are significantly different (LSD, *P* < 0.01). CI, consumption index; AD, approximate digestibility; ECI, efficiency of conversion of ingested food; ECD, efficiency of conversion of digested food; RCR relative consumption rate; RGR, relative growth rate.

**Table 2.**
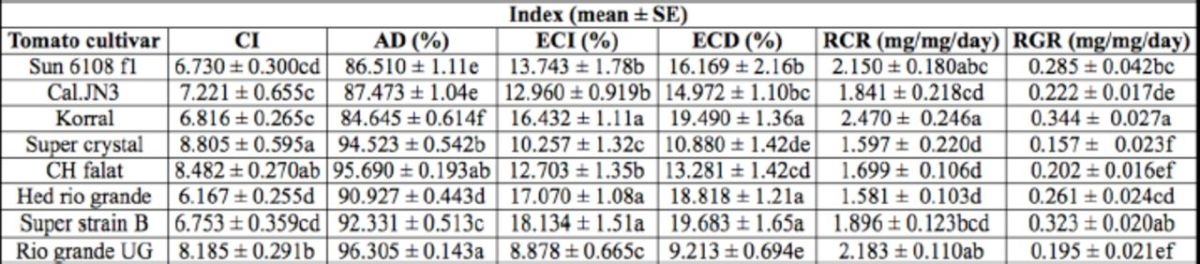
Nutritional indices of fifth instar larvae of *Helicoverpa ar migera* on different tomato cultivars.

The means followed by different letters in the same columns are significantly different (LSD, *P* < 0.01). CI, consumption index; AD, approximate digestibility; ECI, efficiency of conversion of ingested food; ECD, efficiency of conversion of digested food; RCR, relative consumption rate; RGR relative growth rate.

**Table 3.**
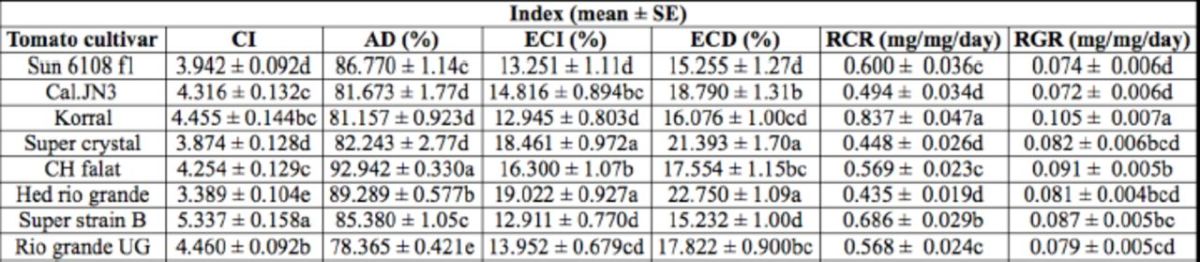
Nutritional indices of sixth instar larvae of *Helicoverpa armigera* on different tomato cultivars.

The means followed by different letters in the same columns are significantly different (LSD, *P* < 0.01). CI, consumption index; of AD, approximate digestibility; ECI, efficiency of conversion of ingested food; ECD, efficiency RCR relative consumption rate; RGR, relative growth rate.

**Table 4.**
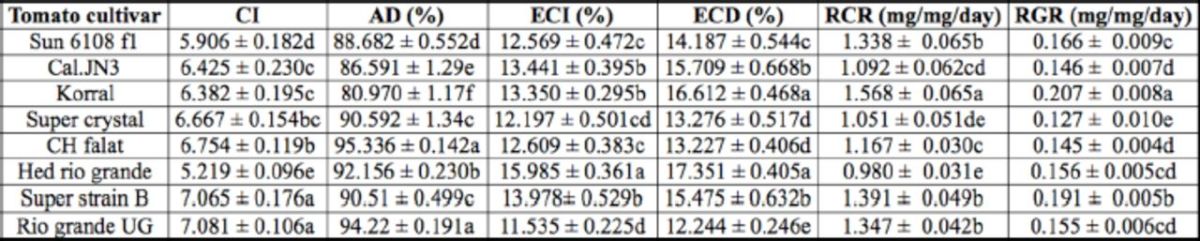
Nutritional indices of whole larval instars of *Helicoverpa armigera* on different tomato cultivars.

The means followed by different letters in the same columns are significantly different (LSD, *P* < 0.01). CI, consumption index; of AD, approximate digestibility; ECI, efficiency of conversion of ingested food; ECD, efficiency RCR relative consumption rate; RGR, relative growth rate.

The highest and lowest CI values ( *F* = 17.10; df = 125; *P* < 0.01) of sixth instar *H. armigera* were respectively on cultivars Super strain B and Hed rio grande. The ECI ( *F* = 6.15; df = 125; *P* < 0.01) and ECD ( *F* = 4.53; df = 125; *P* < 0.01) of larvae reared on Hed rio grande showed the highest values compared to the other cultivars ( Table 3 ).

The results depicted in Table 4 for whole larval instars showed that the ECI ( *F* = 9.49; df = 125; p < 0.01) and ECD ( *F* = 8.02; df = 125; *P* < 0.01) values were the highest on Hed rio grande and lowest on Rio grande UG. The highest and lowest AD values ( *F* = 29.89; df = 125; *P* < 0.01) were respectively on cultivars CH falat and Korral. The larvae that fed on cultivar Korral had the highest RCR ( *F* = 12.55; df = 125; *P* < 0.01) and RGR ( *F* = 10.77; df = 125; *P* < 0.01) values. However, the highest and lowest values of CI were on Rio grande UG and Hed rio grande, respectively ( *F* = 10.46; df = 125; p < 0.01).

**Figure 1.**
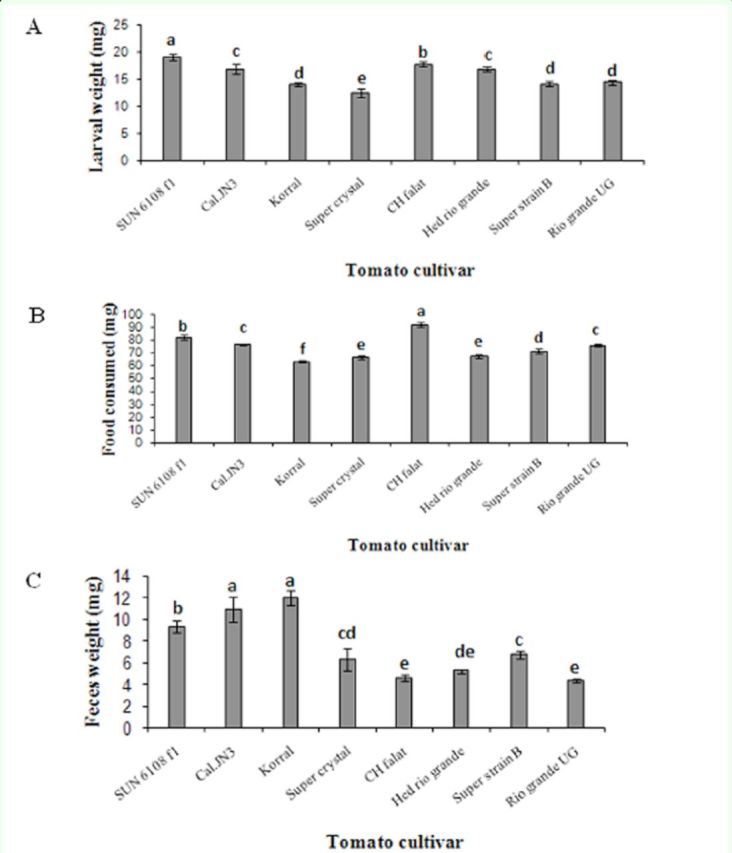
Mean larval weight (A), food consumed (B) and feces produced (C) of whole larval instars of *Helicoverpa armigera* on different tomato cultivars. Bars represent standard error of the means. The means followed by different letters are significantly different(LSD, *P* < 0.01).

The results presented in Fig. 1 for whole larval instars showed significant difference for larval weight (F = 16.84; df = 128; *P* < 0.01), feces produced (F = 21.46; df = 128; *P* < 0.01), and food consumed (F =45.69; df = 128; *P* < 0.01). The highest and lowest values of larval weight were observed on SUN 6108 f1 (18.984 ±0.581 mg) and Super crystal (12.484 ± 0.725 mg), respectively. The food consumed and feces produced were respectively highest on CH falat and Korral (91.910 ± 1.68 and 12.059 ± 0.708 mg, respectively) and lowest on Korral and Rio grande UG (63.422 ± 1.07 and 4.395 ± 0.168 mg, respectively).

**Figure 2:**
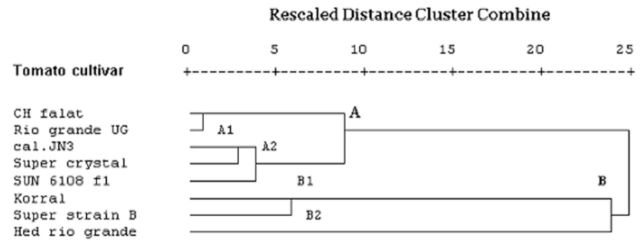
Dendro gram of different tomato cultivars according to nutritional indices of whole larval instars of *Helicoverpa armigera* reared on tomato cultivars (Ward’s method).

A dendrogram based on nutritional indices of whole larval instars of *H. armigera* reared on different tomato cultivars is shown in Fig. 2 . The dendrogram shows two separate clusters labeled A (including subclusters A1 and A2) and B (including subclusters B1 and B2). Different cultivars were grouped within each cluster according to the comparison of the nutritional indices of *H. armigera* fed different tomato cultivars. Cluster A included subclusters A1 (CH falat and Rio grande UG) as a partially resistant group and A2 (Cal.JN3, Super crystal and Sun 6108 f1) as an intermediate group. Cluster B consisted of subclusters B1 (Korral and Super strain B) and B2 (Hed rio grande) as susceptible cultivars.

## Discussion

Study of tomato cultivars for resistance and susceptibility against tomato fruit borer is an important tool to manage the fruit borer with environmentally safe tactics ( Sajjad et al. 2011 ). Differences in allelochemical concentrations, poor nutritional quality of the food, and pericarp thickness between host plant cultivars can affect an insect’s performance as larva and survival and development of insects on different cultivars ( Sharma et al. 1982 ; Samraj and David 1988 ; Martin and Pulin 2004 ). In this study, the data of nutritional indices, especially ECI and ECD values, of *H. armigera* reared on different tomato cultivars were significantly different, which suggests that the cultivars had different nutritional value. Among nutritional indices, ECI is an indicative index of an insect’s ability to utilize the food ingested for growth and development, and ECD is an index of the efficiency of conversion of digested food into growth ( Nathan et al. 2005 ). Also, change in ECD index indicates the overall increase or decrease of the proportion of digested food metabolized for energy ( Naseri et al. 2010 ).

The data generated for the *H. armigera* fifth and sixth instar larvae are inconsistent with each other because the nutritional requirements of the insect change through development, and such differences typically result in changes of food consumption and utilization ( Barton Browne 1995 ). Usually, when the quantity of food ingested is decreased, the duration of development is extended and the insect becomes smaller and lighter. Another reason may be related to increased instar duration, when increased amount of ingested food must be dedicated to maintaining metabolism. Nutritional requirements would be positively correlated with the mass of the insect ( Phillipson 1981 ; Schroeder 1981 ).

The results for the fourth instars showed that the larvae that fed on Cal.JN3 had the highest ECI and ECD values. High ECI and ECD values indicate that the larval feeding and their weight have improved. Consequently, larger pupae are produced, which has a direct correlation to adult fertility, which is ecologically very important for the survival of this insect ( Daryaei et al. 2007 ). Foss and Rieske (2003) suggested that a combination of plant characteristics may be responsible for insect preference and performance, and that an optimal combination of plant components serves to maximize host suitability. Also, as fourth instars, larvae that fed on cultivar CH falat showed the highest AD and almost the lowest ECD. Apparently, the increase in AD value could not compensate for the decrease in ECD value, which consequently led to reduced growth rate. Growth reduction is a general response and reaction of phytophagous insects confronted with a new host plant ( Grabstein and Scriber 1982 ; Sheppard and Friedman 1990 ; Lazarevic and Peric-Mataruga 2003 ).

Higher CI value of the whole *H. armigera* larval instars on Rio grande UG indicated that the rate of intake relative to the mean larval weight during the feeding period was the highest on this cultivar. The results for AD value of the whole larval instars reared on CH falat and Rio grande UG were different from those reported by Hemati et al. (2012) on tomato var. Meshkin (67.470 ± 0.016%). This difference is probably related to difference in tomato cultivars, which are different in acidity and secondary compounds.

Among different tomato cultivars, the highest ECI and ECD values of the whole larval instars were observed on cultivars Hed rio grande and Korral, indicating that they were more efficient at the conversion of ingested and digested food to biomass in larval body. Also, the whole larval instars on Rio grande UG showed the lowest ECD and ECI values, maybe because of a shortage of nutritional components and the existence of some secondary chemicals in this cultivar. The results for ECI values of the larvae that fed on Rio grande UG was nearly similar to those reported by Hemati et al. (2012) on chickpea (var. Arman) (11. 981 ± 0.006%). The mean ECD value of whole larval instars reared on different tomato cultivars was lower than that reported by Naseri et al. (2010) on soybean cultivars (60.592 ± 2.012%), suggesting that the larvae that fed on fruit of tomato cultivars were apparently not efficient in turning digested food into biomass compared with those that fed on pods of soybean cultivars. This finding related to the unsuitability of tomato agrees with the results of Liu et al. (2004) , who reported that tomato was an unsuitable host plant compared with other test hosts.

For the whole larval instars, the larval weight, as an important fitness indicator of insect population dynamics ( Liu et al. 2004 ), was highest on SUN 6108 f1 and CH falat. This reinforces the suggestion that SUN 6108 f1 and CH falat are more suitable cultivars for *H. armigera* larvae than the others. Also, the larvae reared on cultivar Super crystal were lighter than those reared on the other cultivars. We found that the larval weight of *H. armigera* reared on cultivar Korral was similar to that reported by Hemati et al. (2012) on tomato var. Meshkin (15.205 ± 0.272 mg) and Naseri et al. (2010) on soybean var. ‘L17’ (15.497 ± 0.911 mg).

The duration of developmental period of larval stages is a determining factor that shows whether a cultivar is suitable or unsuitable for feeding larvae, as the lepidopteran larvae fed highly-nutritious food increase growth rates and complete development period faster than those larvae that feed on low-nutrient food ( Hwang et al. 2008 ). For the RGR and RCR values, the duration of feeding period is an effective factor. Among different tomato cultivars, the highest RCR and RGR values of the whole larval instars *H. armigera* were on cultivar Korral and the lowest ones were on Hed rio grande and Super crystal. Our results indicate that Hed rio grande and Super crystal have low nutritive value for larvae of *H. armigera,* and on these cultivars a longer development period was needed to complete immature stages. Conversely, the cultivar Korral was highly nutritive for the larvae, and a shorter period of development was necessary to complete immature stages.

Comparing the results of Tables 1-3 shows that the ECI and ECD values, in most cases, increased from fourth to sixth (ultimate) instar. The trend of increase in ECD from early to late instars has been reported by Slansky and Scriber (1985) and Hemati et al. (2012) . Nation (2001) noted that the physiological changes in the penultimate and ultimate instars reared on different host plants probably are responsible for the differences in such decreases in ECD values of the two larval instars.

High ECI and ECD values were frequently associated with low CI and vice versa. A similar result was reported in numerous studies on *H. armigera* and other insects ( Barbosa and Greenblatt 1979 ; Naseri et al. 2010 ; Arghand et al. 2011 ; Hemati et al. 2012) . Also, the results of whole larval instars showed that there is a negative correlation between RCR and ECI values. Soo Hoo and Fraenkel (1966) noted that the negative correlations between RCR and ECI could have either of two explanations. First, when larvae consume less, the food tends to pass through their digestive system more slowly, and so it can be converted more completely and used by the insects. Second, it may be that insects consume less of a special food simply because they are capable of converting it more efficiently and therefore do not need to eat large quantities of that food to reach appropriate levels of growth.

The results of the cluster analysis revealed that grouping within each cluster might be due to a high level of physiological similarity of different tomato cultivars. The results of the comparative nutritional indices of *H. armigera* on different tomato cultivars indicated that subcluster A1 was the least suitable, subclusters B1 and B2 were the most suitable cultivars for *H. armigera,* and the cultivars in subcluster A2 had an intermediate status.

The results of this study suggest that cultivars Korral and Super strain B are more nutritive, and cultivars CH falat and Rio grande UG are less nutritive for *H. armigera* larvae compared with the other test cultivars .

Analysis of nutritional indices can lead to the understanding of the behavioral and physiological basis of an insect’s response to host plants ( Lazarevic and Peric-Mataruga 2003 ). The lesser suitability of some cultivars as host plants of *H. armigera* may be due to the presence of some secondary phytochemicals in these cultivars or the absence of primary nutrients necessary for growth and development of *H. armigera.* These findings will be helpful in understanding the host preference of this particular pest and could help in its management and control, particularly on tomato. Therefore, future studies should focus on testing a wider range of cultivars of tomato for the nutritional indices of *H. armigera.* Finally, assessment of the chemical components of the test cultivars would be helpful to better understand the mechanism of host suitability.
